# An exploration of changes in plantar pressure distributions during walking with standalone and supported lateral wedge insole designs

**DOI:** 10.1186/s13047-021-00493-5

**Published:** 2021-10-06

**Authors:** Calvin T. F. Tse, Michael B. Ryan, Jason Dien, Alex Scott, Michael A. Hunt

**Affiliations:** 1 Motion Analysis and Biofeedback Laboratory, University of British Columbia, Vancouver, BC Canada; 2 Graduate Programs in Rehabilitation Sciences, University of British Columbia, Vancouver, BC Canada; 3 Kintec Footlabs Inc, Surrey, BC Canada; 4 Department of Biomedical Physiology and Kinesiology, Simon Fraser University, Burnaby, BC Canada; 5 Department of Physical Therapy, University of British Columbia, Vancouver, BC Canada

**Keywords:** Plantar pressure, Pressure distribution, Lateral wedge insole, Supported lateral wedge

## Abstract

**Background:**

Lateral wedge insoles (LWI), standalone or with medial arch support (supported-LWI), have been thoroughly investigated for their effects on modifying gait biomechanics for people with knee osteoarthritis. However, plantar pressure distribution between these insole types has not been investigated and could provide insight towards insole prescription with concomitant foot symptoms taken into consideration.

**Methods:**

In a sample of healthy individuals (*n* = 40), in-shoe plantar pressure was measured during walking with LWI, with or without medial arch support (variable- and uniform-stiffness designs), and a flat control insole condition. Pressure data from the plantar surface of the foot were divided into seven regions: medial/lateral rearfoot, midfoot, medial/central/lateral forefoot, hallux. Plantar pressure outcomes assessed were the medial-lateral pressure index (MLPI) for the whole foot, and the peak pressure, pressure-time integral (PTI), and contact area in each plantar region. Comfort in each insole condition was rated as a change relative to the flat control insole condition. Repeated-measures analyses of variance were calculated to compare the plantar pressure outcomes between insole conditions.

**Results:**

Regionally, medial rearfoot and forefoot pressure were reduced by all wedged insoles, with the variable-stiffness supported-wedge showing greater reductions than the standalone wedge. Lateral rearfoot and forefoot pressure were reduced by both supported-LWI, but unchanged by the standalone wedge. In the midfoot, the standalone wedge maintained pressure but reduced regional contact area, while both supported-LWI increased midfoot pressure and contact area. All LWI increased the MLPI, indicating a lateral shift in plantar pressure distribution throughout the weightbearing phase of gait. Comfort ratings were not significantly different between insole conditions.

**Conclusions:**

Regional differences in plantar pressure may help determine an appropriate lateral wedge insole variation to avoid exacerbation of concomitant foot symptoms by minimizing pressure in symptomatic regions. Lateral shifts in plantar pressure distribution were observed in all laterally wedged conditions, including one supported-LWI that was previously shown to be biomechanically ineffective for modifying knee joint load distribution. Thus, shifts in foot centre of pressure may not be a primary mechanism by which LWI can modify knee joint load distribution for people with knee osteoarthritis.

**Supplementary Information:**

The online version contains supplementary material available at 10.1186/s13047-021-00493-5.

## Background

Shoe-worn lateral wedge insoles (LWI) are simple tools used to modify gait biomechanics in people with medial tibiofemoral osteoarthritis (knee OA). In this clinical population, conservative biomechanical interventions typically target reductions in magnitudes of the knee adduction moment (KAM), a surrogate of knee load distribution linked to structural [1, 2] and clinical worsening of knee OA [3]. LWI, as standalone insoles or combined with medial arch supports (supported-LWI), have been thoroughly investigated for their effect on the KAM, with a recent systematic review and meta-analysis reporting 5–10% reductions across various outcomes of the KAM during walking with various LWI designs [4]. As a foot-based intervention, evaluating LWI for their effects on modifying plantar pressure distribution would help inform other clinically-relevant features, such as regional loading and comfort.

There is growing interest in concomitant foot and ankle symptoms and their link to clinical features of knee OA. Recent analyses of the Osteoarthritis Initiative database have highlighted relationships between symptomatic knee OA and concomitant foot and ankle symptoms, defined as pain, aching, or stiffness for more than half of the days in the past 30 days [5–7]. Patients with knee OA and concomitant foot symptoms have exhibited poorer outcomes of overall health and physical function at baseline [5], and a higher risk of worsening knee pain in the subsequent 4 years [7], compared to knee OA patients without concomitant foot symptoms. Further, in individuals at risk of developing knee OA, but asymptomatic and without radiographic signs at baseline, the presence of foot and ankle symptoms increased the likelihood of developing knee pain and radiographic signs of OA over 4 subsequent years [6]. A consistent effect of LWI is increased ankle eversion and external eversion moment demands [4, 8, 9], with greater angles of wedging also negatively affecting self-reported comfort [10, 11]. Supported-LWI have been shown to be effective at minimizing ankle eversion effects, while still reducing the KAM [8, 9, 12], and may be preferred over a standalone LWI [13]. Considering the clinical concerns of concomitant foot symptoms in knee OA and that multiple LWI options have demonstrated biomechanical efficacy, selecting an insole for patients to avoid exacerbation of existing foot symptoms appears to be paramount.

Evaluations of pressure distribution underfoot with LWI have primarily reported lateral shifts in the centre of pressure (CoP) [9, 14, 15]. Lateralization of CoP is hypothesized to reduce the KAM by shifting the medially-oriented ground reaction force to shorten the frontal plane moment arm between the reaction force vector and the knee joint. Shifts in CoP may not be a reliable mechanism of KAM reduction, however, since medial shifts in CoP have also been found to reduce the KAM, as seen with medial thrust gait [16] and laterally-wedged footwear (wedged externally on the outsole) [17]. Investigations of KAM reduction often report CoP measured by floor-mounted force platforms, which provide high fidelity kinetic information about the interface between footwear and the ground, necessary for calculating joint moments. In comparison, flexible in-shoe pressure sensors provide a less expensive and portable method of measuring pressure between the plantar foot surface and the insole, in particular those with non-planar designs. With a method for plantar pressure assessment that is feasible for clinical settings, relevant information about the foot-insole interface could inform insole prescription when concomitant foot symptoms are present.

Assessments of regional plantar pressure distribution could provide insight into how redistribution of plantar pressure with insoles could influence perceived symptoms. In people experiencing foot pain from work-related prolonged standing, contoured insoles shifted regional peak pressures from the rearfoot to the midfoot, and reduced sensations of pain, discomfort, and fatigue compared to not wearing the insoles [18]. A cross-sectional exploration of patients with knee OA reported that greater knee pain was associated with increased medial plantar loading and midfoot contact area during gait [19], which suggests plantar pressure distribution could be linked to perceived symptoms in the lower limb with knee OA. In-shoe measured plantar pressure effects for varus and valgus wedged insoles [20] and externally-wedged footwear [21] have reported lateral shifts in pressure with lateral foot posting. However, the regional plantar pressure effects have not been evaluated for supported-LWI, nor interpreted with the intent of informing insole treatment amongst concomitant foot symptoms. Therefore, the aim of this study was to explore the effects of standalone LWI and supported-LWI on plantar pressure distribution and perceived comfort during gait in healthy adults.

## Methods

The current investigation is a complementary analysis of plantar pressure and comfort data obtained during gait analysis of various LWI designed for knee OA. A summary of the joint kinematic and kinetic outcomes for these insoles were reported in Tse et al. [12], which found standalone medial arch supports increased the KAM. Similar findings of increased KAM with medial arch supports have been reported [22, 23], despite Hinman et al. [23] reporting non-significant increases in a small sample with large variability in KAM change. For this reason, standalone medial arch supports were excluded from the current analysis of plantar pressure outcomes. However, outcomes across all insole conditions are reported in Appendix 1 of supplementary materials. Additionally, the current sample size was justified for detecting a change in KAM with LWI from the affiliated study [12]. Therefore, an additional sample size justification was not conducted for the current exploratory assessment of plantar pressure distribution and perceived comfort with standalone LWI and supported-LWI.

### Participants

A convenience sample of healthy adults from the university and surrounding community were recruited via electronic and print media and word of mouth. Exclusion criteria for study participation included any history of orthotic insole use in the 12 months prior to physical screening, or any musculoskeletal or neurological condition that impaired gait at the time of testing. Ethics approval for this study was received from the institutional Clinical Research Ethics Board. All participants received written and verbal explanations of the details prior to providing written consent for study enrolment.

### Orthotic insoles

**Fig. 1:**
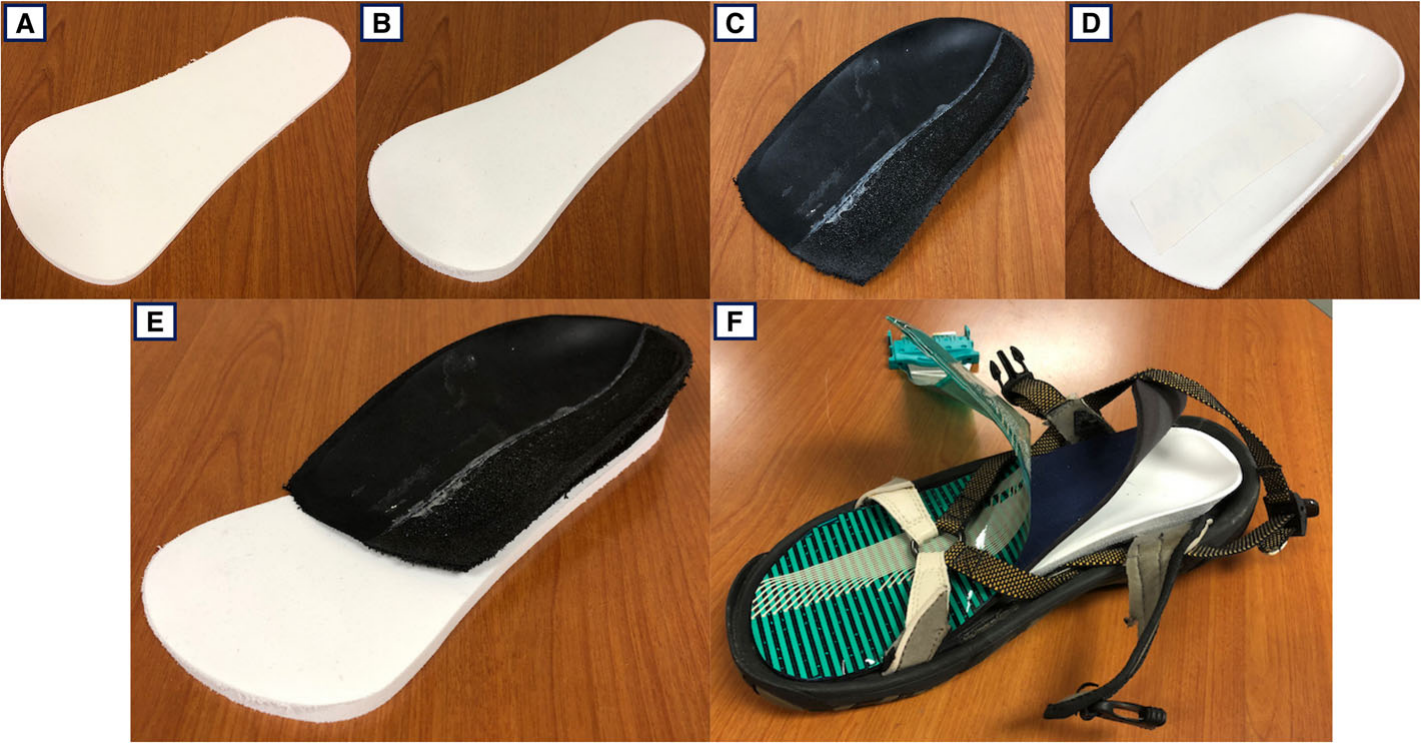
Insoles and standardized sandal set-up used for walking trials. **A** 3 mm flat control (FLAT). **B** 5° lateral wedge (WEDG). **C** Variable-stiffness arch support (V-ARCH). **D** Uniform-stiffness arch support (U-ARCH). **E** Example of supported lateral wedge (WEDG+V-ARCH shown). **F** Sandal setup with insole, neoprene topcover, and plantar pressure sensor

Four pairs of sulcus length orthotics were custom-fabricated for each participant, using three-dimensional laser volumetric casting by a Canadian Board-Certified Pedorthist (Fig. 1). Non-contoured insoles fabricated from ethyl-vinyl acetate foam (EVA) (Shore A stiffness 55) included a neutral 3 mm flat control (FLAT) and 5° lateral wedge (WEDG). Two pairs of custom contoured arch support insoles were fabricated from the volumetric casts: (1) variable-stiffness (V-ARCH) was constructed with plastazote foam laterally (Shore A stiffness 70) and EVA medially (Shore A stiffness 20), (2) uniform-stiffness (U-ARCH) was constructed with EVA (Shore A stiffness 55). Two supported-LWI conditions were created by affixing each custom arch support to the top of the WEDG: WEDG+V-ARCH and WEDG+U-ARCH. All insoles were covered with a full-length piece of neoprene and secured into a standardized sandal during all walking trials (Fig. 1).

### Procedure

All participants were randomly assigned a study limb of interest and fitted with standardized sandals to match their foot dimensions. The sandals had a neutral heel to toe drop, Velcro straps to secure them to the feet, and removeable footbeds, into which orthotic insoles could be fitted. Prior to collecting data during walking in each insole condition, participants were encouraged to acclimate to the insole and sandals by walking freely and resolving any fit abnormalities. For all participants the FLAT condition was tested first, and the five remaining insole conditions were systematically randomized with a Williams Design to account for possible order and carry-over effects. Walking speed was measured using two commercial photoelectric timing gates placed a known distance apart, and self-selected walked speed was established as the mean walking speed during walking trials with FLAT. Walking trials were deemed successful if the speed was within 5% of the average walking speed with FLAT for all other conditions, and if the study limb foot struck the ground completely within the boundary of a floor-embedded force platform. A minimum of five successful walking trials along a 10 m walkway were recorded in each insole condition.

### Data collection and reduction

Plantar pressure was measured bilaterally using flexible shoe-embedded sensors (F-Scan, Tekscan, Boston, MA, USA) at 100 Hz. All sensors were trimmed to match the sandal size and secured to the neoprene topcover with double-side tape to ensure consistent sensor placement among insole conditions (Fig. 1). Each sensor detects pressure as an array of sensing units, with a surface area resolution of 0.258 cm^2^ per sensing unit. Only data collected during the stance phase of gait were processed and analyzed. For each calculated outcome, the average of five successful walking trials was calculated to represent its value in each insole condition.

**Fig. 2:**
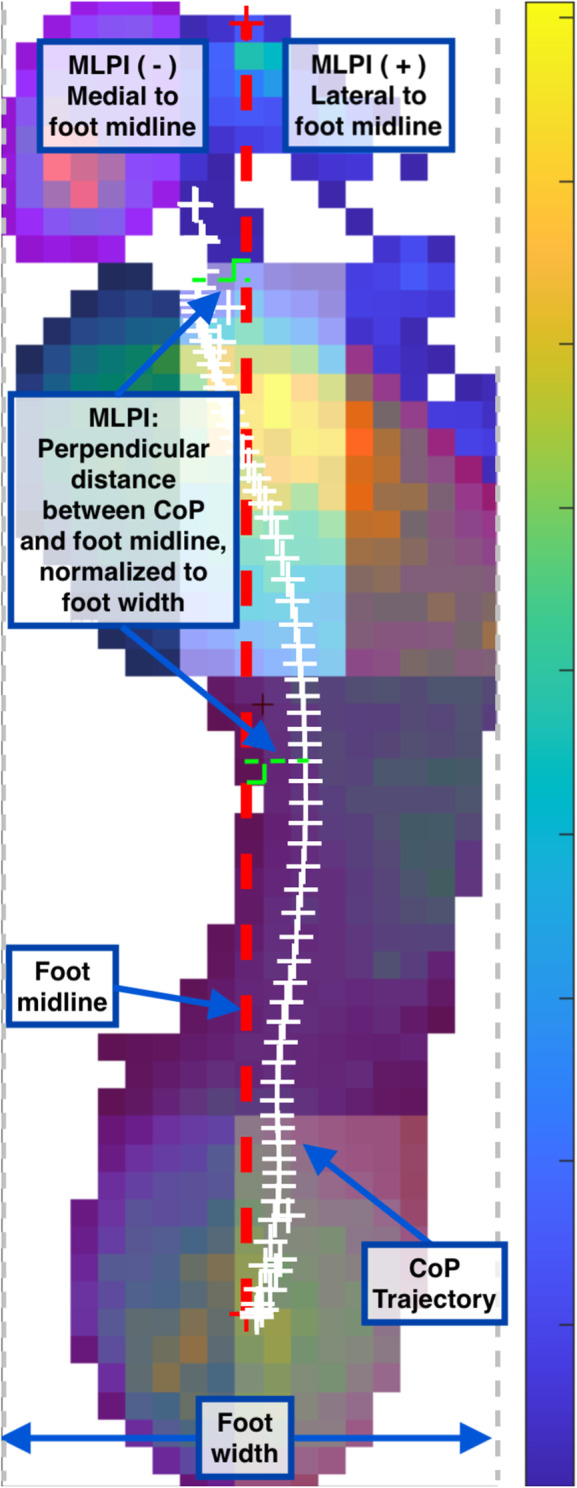
Representative plantar pressure map during a single stance phase. The coloured box overlays demarcate the complete pressure map into multiple plantar regions used for calculating pressure outcomes. Red dashed line between the heel centroid and second toe delineates the midline of the foot. White crosses (+) denote the trajectory of the centre of pressure (CoP) throughout stance phase. Green dashed lines demonstrate how the time-series medial-lateral pressure index is calculated as the distance between the CoP and the foot midline (normalized to foot width). Positive and negative MLPI indicate a CoP that is lateral and medial to the foot midline, respectively

Pressure data recorded by each sensing unit was filtered using a zero-lag 4th order low-pass Butterworth filter with a 25 Hz cut-off. For faulty sensing units that detected non-physiological pressure, its data were replaced with the time-series average of its eight neighbouring sensing units. A semi-automated masking program was used to segment the foot into seven plantar regions: hallux, forefoot (medial, central, lateral), midfoot, rearfoot (medial, lateral) (Fig. 2). All masks were visually checked, and any erroneous regional masks were manually corrected. For each plantar region, a representative time-series pressure signal was calculated as the average pressure of all active sensing units within the region. From the regional time-series pressure data, the discrete outcomes extracted for analysis included: peak pressure (kPa), pressure-time integral (kPa*sec), and time of peak pressure (% stance). The contact area in each plantar region was calculated as the sum of active sensing units in the region multiplied by the surface area per unit.

The medial-lateral pressure index (MLPI) represents the medial-lateral plantar pressure distribution with respect to the midline of the foot (heel centroid to 2nd toe) throughout stance phase (Fig. 2). For each frame of data, MLPI was calculated as the perpendicular distance between the centre of pressure (CoP) and the midline of the foot, normalized to the foot width [24]. Positive and negative values indicate a CoP that is lateral or medial to the midline of the foot, respectively. For each walking trial, the mean MLPI and area under the MLPI curve (AUC) during the first and second halves of stance phase were calculated separately.

Participants rated insole comfort on a 15-point global rating of change scale [25]. Using the FLAT insole condition as the reference comparator, + 7 and − 7 represented a change in comfort that was maximally “improved” or “reduced”, respectively. Zero represented no change in comfort (equivalent to FLAT). Following completion of walking trials in all insole conditions, participants selected one insole as their most preferred.

### Statistical analysis

Normality was evaluated via visual inspection of histograms and supplemented with a Shapiro-Wilk test. Homogeneity of variance was evaluated via Mauchly’s test of sphericity. For discrete outcomes of MLPI, regional plantar pressure, and comfort change, repeated measures analyses of variance were used to test for differences between four insole conditions (FLAT, WEDG, WEDG+V-ARCH, WEDG+U-ARCH). Significant main effects of insole condition were followed-up with post hoc Tukey’s HSD pairwise comparisons. Statistical calculations were completed with jamovi version 1.6 [26], at an alpha level of α = 0.05.

## Results

Forty healthy individuals participated in the study with the following demographic information: 23 males, 17 females, mean (SD) age = 26.6 (2.9) years, height = 173.5 (8.6) cm, body mass = 71.2 (12.7) kg, BMI = 23.5 (2.8) kg/m^2^. The median foot posture index was = 5 (25th percentile = 2; 75th percentile = 9). All evaluated outcomes satisfied the assumptions for normality and homogeneity of variance. The following summarizes the outcomes for the four conditions that were statistically compared (FLAT, WEDG, WEDG+V-ARCH, and WEDG+U-ARCH). An extended summary of discrete outcomes for all insoles can be found in Appendix 1 of supplementary materials.

### Regional plantar pressure

#### Lateral regional pressure

**Fig. 3:**
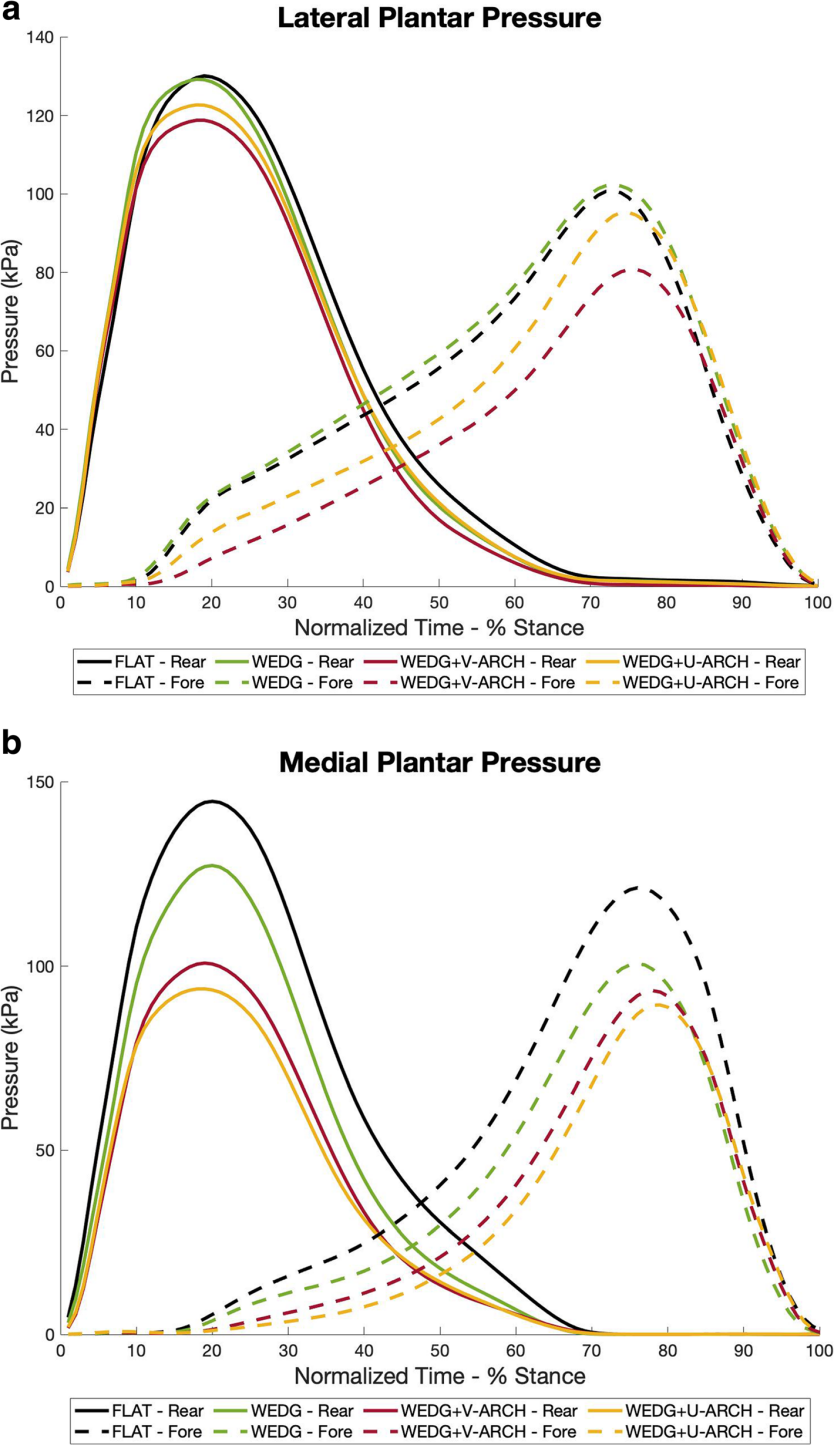
Ensemble average of the regional plantar pressure for the (**a**) lateral and (**b**) medial rearfoot (solid lines) and forefoot (dashed lines). The colours representing each insole condition are as follows: FLAT (black), WEDG (green), WEDG+V-ARCH (red), WEDG+U-ARCH (yellow)

Lateral rearfoot and forefoot peak pressure and pressure-time integral were decreased with WEDG+V-ARCH (*p* < 0.05), when compared to FLAT. WEDG+V-ARCH also had lower peak pressure and pressure-time integral compared to WEDG (*p* < 0.05). WEDG+U-ARCH reduced the peak pressure in the lateral rearfoot and the pressure-time integral in lateral forefoot, compared to FLAT (both *p* < 0.05). Discrete plantar pressure outcomes for all regions are reported in Table 1, and ensemble average curves for the lateral rearfoot and forefoot regions are found in Fig. 3a.

**Table 2:** Medial-Lateral Pressure Index (MLPI) outcomes by insole condition

MLPI Outcome		FLAT	WEDG	WEDG + V-ARCH	WEDG + U-ARCH
Early Stance	Mean (% foot width)	3.2 (2.9)	5.5 (2.5) ^**a**^	5.8 (3.0) ^**a**^	6.9 (3.0) ^**a,b**^
	AUC (% foot width ^*^ sec)	1.0 (0.9)	1.8 (0.8) ^a^	1.9 (1.0) ^**a**^	2.3 (1.0) ^**a,b**^
Late Stance	Mean (% foot width)	−2.5 (5.7)	0.5 (6.0) ^a^	−0.4 (5.9) ^**a**^	0.9 (6.2) ^**a**^
	AUC (% foot width ^*^ sec)	−0.8 (1.9)	0.2 (2.0) ^**a**^	−0.1 (2.0) ^**a**^	0.4 (2.1) ^**a**^

All values reported as mean (standard deviation)

Positive (lateral) and negative (medial) values indicate the centre of pressure position relative to the midline of the foot

^a^ denotes a significant difference from FLAT (*p* < 0.05)

^b^ denotes a significant difference from WEDG (*p* < 0.05)

Contact area in the lateral rearfoot was increased with WEDG+V-ARCH and WEDG+U-ARCH compared to FLAT (*p* < 0.05) and WEDG (*p* < 0.05). Lateral forefoot contact area decreased with supported-LWI WEDG+V-ARCH and WEDG+U-ARCH compared to FLAT (*p* < 0.05). WEDG did not significantly change contact area in the lateral rear- or forefoot compared to FLAT (*p* ≥ 0.06). Discrete contact area values for all regions are reported in Table 1.

#### Medial regional pressure

Medial rearfoot and forefoot peak pressure and pressure-time integral were reduced by WEDG, WEDG+V-ARCH, and WEDG+U-ARCH (all *p* < 0.05) when compared to FLAT. Both supported-LWI (WEDG+V-ARCH and WEDG+U-ARCH) also reduced medial rearfoot and forefoot peak pressure and pressure-time integral compared to WEDG (*p* < 0.05). Ensemble average curves for the medial rear- and forefoot regions are found in Fig. 3b.

Contact area in the medial rearfoot decreased with WEDG, compared to FLAT (*p* < 0.05). The WEDG+V-ARCH and WEDG+U-ARCH increased medial rearfoot contact area when compared to WEDG (*p* < 0.05), but were not different from FLAT. Medial forefoot contact area was unchanged by any insole compared to FLAT (*p* ≥ 0.32).

#### Midfoot pressure

**Fig. 4:**
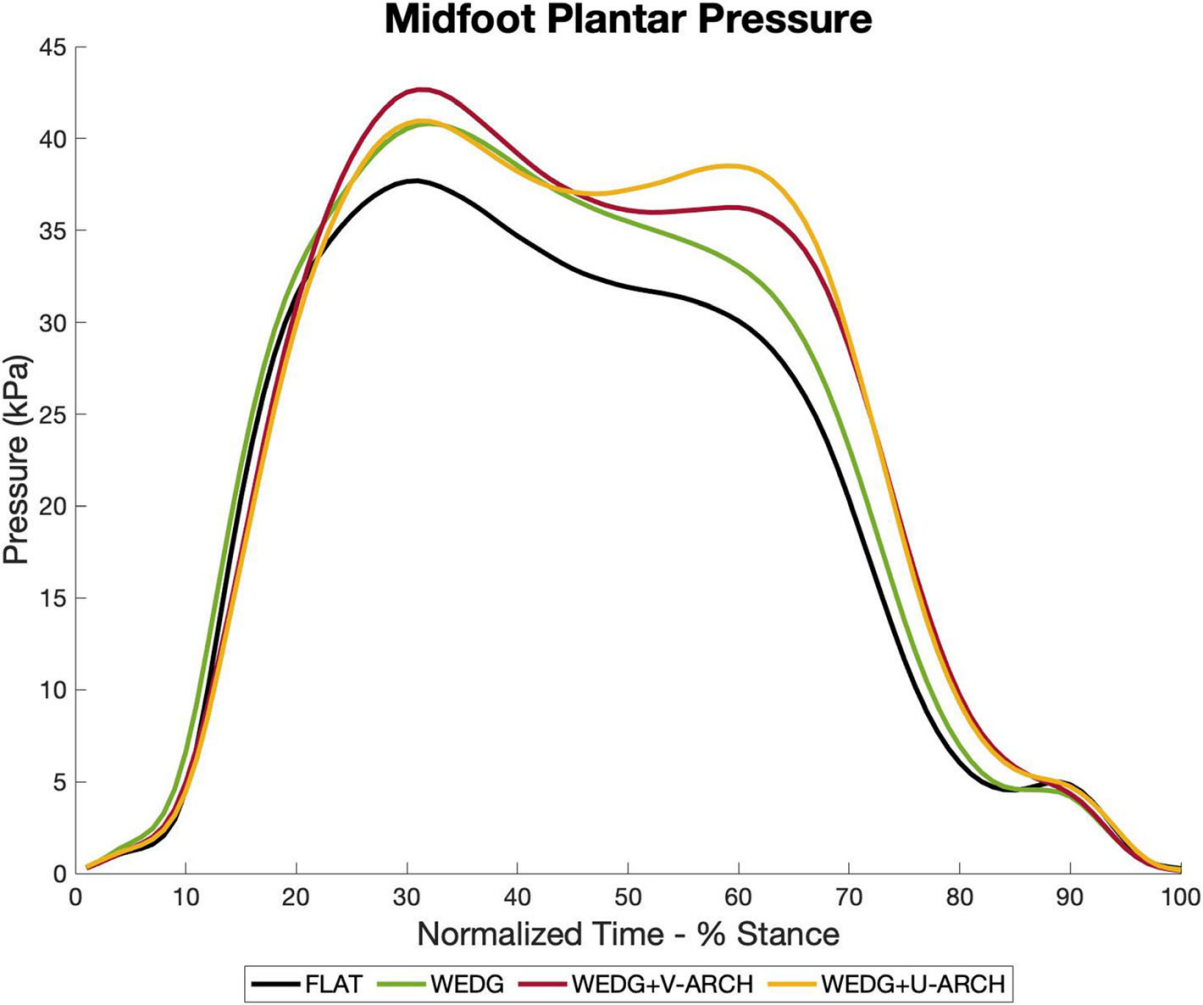
Ensemble average of the regional plantar pressure for the midfoot. The colours representing each insole condition are as follows: FLAT (black), WEDG (green), WEDG+V-ARCH (red), WEDG+U-ARCH (yellow)

Midfoot peak pressure and pressure-time integral were unchanged with WEDG (*p* ≥ 0.13), and increased with WEDG+V-ARCH (*p* < 0.05) and WEDG+U-ARCH (*p* < 0.05), compared to FLAT. Ensemble average curves for the region are found in Fig. 4.

Compared to FLAT, midfoot contact area was increased with WEDG+V-ARCH and WEDG+U-ARCH (*p* < 0.05), while decreased with WEDG (*p* < 0.05).

### Medial-lateral pressure distribution

**Fig. 5:**
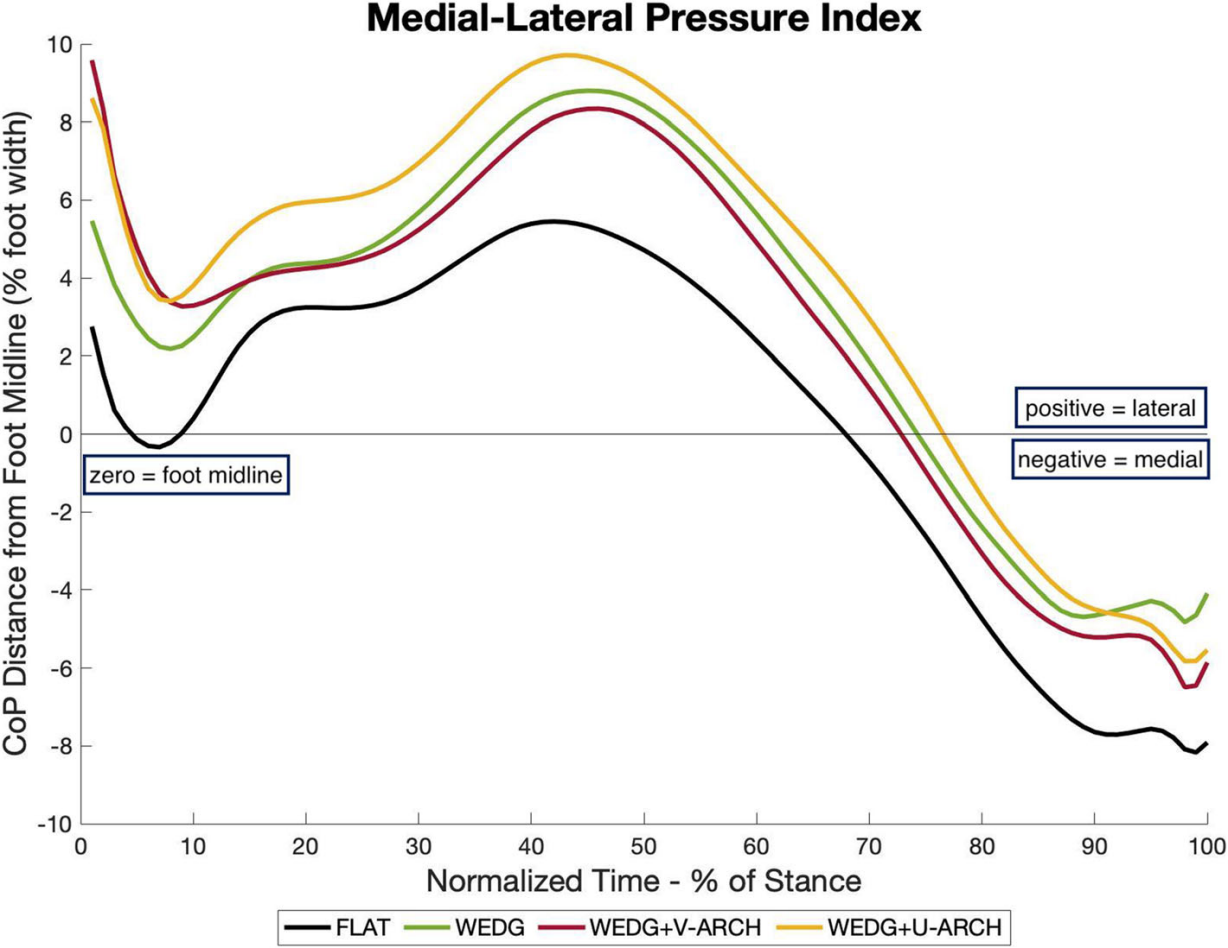
Ensemble average of the medial-lateral pressure index throughout stance phase for all insole conditions. Positive and negative MLPI indicate a CoP that is lateral or medial to the foot midline, respectively. The colours representing each insole condition are as follows: FLAT (black), WEDG (green), WEDG+V-ARCH (red), WEDG+U-ARCH (yellow)

Through early and late stance phase, all laterally-wedged insole conditions (WEDG, WEDG+V-ARCH, WEDG+U-ARCH) shifted the medial-lateral plantar distribution laterally, exhibiting increased mean and AUC MLPI compared to FLAT (*p* < 0.05). Discrete values of MLPI are summarized in Table 2 and their corresponding ensemble average curves for all insole conditions are found in Fig. 5.

**Table 1:** Regional plantar pressure outcomes by insole condition, reported as mean (standard deviation)

Plantar Region	Pressure Outcome	FLAT	WEDG	WEDG + V-ARCH	WEDG + U-ARCH
Lateral Rearfoot	Peak Pressure (kPa)	133.0 (24.1)	132.6 (26.0)	122.0 (22.6) ^a,b^	126.1 (23.4) ^a^
	Pressure-Time Integral (kPa*sec)	30.0 (6.4)	29.0 (5.3)	26.7 (5.5) ^a,b^	28.3 (6.9)
	Contact Area (cm^2^)	18.7 (2.3)	18.1 (2.1)	19.5 (2.2) ^a,b^	19.9 (2.6) ^a,b^
Lateral Forefoot	Peak Pressure (kPa)	106.1 (31.8)	108.6 (33.3)	85.6 (26.7) ^a,b^	100.7 (32.2)
	Pressure-Time Integral (kPa*sec)	30.6 (10.4)	32.4 (10.7)	22.0 (7.3) ^a,b^	26.8 (10.1) ^a,b^
	Contact Area (cm^2^)	12.9 (2.6)	12.6 (2.3)	12.2 (2.2) ^a^	12.3 (2.2) ^a^
Medial Rearfoot	Peak Pressure (kPa)	150.3 (23.2)	131.9 (26.1) ^a^	105.3 (27.6) ^a,b^	99.3 (21.5) ^a,b^
	Pressure-Time Integral (kPa*sec)	32.9 (6.7)	26.6 (5.8) ^a^	21.4 (6.1) ^a,b^	20.4 (5.2) ^a,b^
	Contact Area (cm^2^)	17.5 (2.1)	16.5 (2.1) ^a^	17.9 (2.5) ^b^	17.9 (2.8) ^b^
Medial Forefoot	Peak Pressure (kPa)	125.3 (43.0)	105.4 (39.6) ^a^	96.9 (34.2) ^a,b^	93.4 (37.0) ^a,b^
	Pressure-Time Integral (kPa*sec)	30.1 (10.3)	23.4 (8.6) ^a^	20.2 (7.3) ^a,b^	18.3 (7.6) ^a,b^
	Contact Area (cm^2^)	14.4 (1.8)	13.9 (2.1)	14.5 (2.6)	14.7 (2.6)
Midfoot	Peak Pressure (kPa)	42.8 (11.8)	45.6 (10.6)	48.1 (12.9) ^a^	48.0 (12.9) ^a^
	Pressure-Time Integral (kPa*sec)	14.1 (4.6)	15.5 (4.7)	16.2 (4.8) ^a^	16.2 (5.2) ^a^
	Contact Area (cm^2^)	34.7 (8.7)	31.8 (7.7) ^a^	50.9 (9.3) ^a,b^	52.8 (9.1) ^a,b^

All values reported as mean (standard deviation)

^a^ denotes a significant difference from FLAT (*p* < 0.05)

^b^ denotes a significant difference from WEDG (*p* < 0.05)

### Comfort change

Rated as a change in comfort compared to FLAT, the comfort ratings for WEDG (mean [SD] = − 1.2 [2.3]), WEDG+V-ARCH (mean [SD] = − 0.8 [2.3]), and WEDG+U-ARCH (mean [SD] = − 0.7 [2.5]) were not significantly different from each other (*p* ≥ 0.58).

## Discussion

In the current study we assessed plantar pressure distributions of healthy individuals walking with LWI and supported-LWI compared to a flat control insole. The regional distribution of plantar pressure differed between insole conditions. WEDG reduced medial rearfoot and forefoot pressure, while maintaining lateral rearfoot and forefoot pressures compared to FLAT. Meanwhile, WEDG+V-ARCH exhibited greater reductions in medial and lateral rearfoot and forefoot pressure than WEDG, and increased midfoot pressure and contact area compared to FLAT. Early and late stance phase MLPI were increased by all wedged insole conditions, indicative of a lateral shift in CoP throughout the stance phase of gait. No differences in comfort were observed between insole conditions. In light of the redistribution of plantar pressure between LWI conditions, insole prescription may be best informed by its appropriateness for pre-existing foot symptoms. Especially in patients with concomitant knee OA and foot symptoms, clinicians incorporating insoles for biomechanical intervention of medial tibiofemoral OA should consider both kinetic effects at the knee and effects on pressures in plantar regions in their clinical decision making.

A speculated mechanism by which LWI reduce the KAM is through lateralization of the foot CoP, which reduces the frontal plane moment arm between the knee joint and the medially-oriented ground reaction force vector. We found the LWI and supported-LWI increased the mean and AUC of MLPI by 1–4% of foot width (~ 1–4 mm) throughout stance phase, which is in agreement with the lateral shifts in CoP reported by previous works that also evaluated gait with and without LWI, but that used force platform technology [9, 14, 15]. While CoP lateralization appears to be involved in KAM reduction with LWI, it is unlikely to be the primary mechanism of effect. Hinman et al. reported only a weak correlation (*r* = 0.25) between the lateral shift in CoP with LWI and change in KAM peak [15]. In the current study we also found WEDG+U-ARCH had the largest lateral shifts in CoP, but was previously reported to be ineffective at reducing the KAM [12]. In a different biomechanical assessment of medial thrust gait, a medial shift in CoP was found to be linearly related to KAM reduction (*r* = 0.40–0.70) [16]. Considering these discrepant shifts in foot CoP and KAM reduction, it is possible lateralizing CoP with LWI is less important to reducing the KAM than other alterations occurring between the foot and knee, such as frontal plane tibial inclination or knee angle. The frontal plane knee alignment during gait has been shown to completely mediate the relationship between ankle eversion and magnitudes of the KAM [27], which is relevant to how LWI can alter KAM as a foot-based biomechanical intervention. It is important to acknowledge our investigation measured CoP with flexible in-shoe sensors atop non-planar insole surfaces, which may differ from CoP measured by floor-mounted force plates. Nonetheless, the value of in-shoe plantar pressure assessment of LWI may be more relevant to considerations regarding regional pressure changes that could impact comfort or existing foot symptoms.

The growing concern between concomitant foot and ankle symptoms and poorer clinical features of knee OA [5, 7] highlights a need for LWI intervention to minimize the likelihood of generating or exacerbating foot symptoms. Our affiliated evaluation of the insoles from this study found WEDG and WEDG+V-ARCH to be effective at KAM reduction [12]. Differences in the regional plantar pressure profiles may help inform which of these two LWI could be more appropriate for biomechanical intervention in patients experiencing concomitant foot symptoms. For example, in the WEDG condition, peak pressure and PTI were maintained in the lateral rearfoot and forefoot, but reduced in the medial rearfoot and forefoot, compared to FLAT. Meanwhile, WEDG+V-ARCH exhibited greater reductions in the medial and lateral rearfoot and forefoot than WEDG, with lower peak pressure and PTI (Fig. 3 & Table 1). The arch contouring and variable-stiffness design of WEDG+V-ARCH appeared to be redistributing pressure from the rearfoot and forefoot into the midfoot, which was seen as greater midfoot peak pressure, PTI, and contact area compared to FLAT (Fig. 4 & Table 1). Previously, increased medial and decreased lateral peak foot forces during walking have been associated with greater self-reported knee pain in symptomatic patients with knee OA [19]. As such, the observed pattern for WEDG and WEDG+V-ARCH to redistribute plantar pressure laterally by reducing medial pressures may be relevant to the treatment of medial knee OA. Selecting a LWI to minimize regional pressures based on patients’ existing foot symptoms may also be less likely to elicit adverse reactions to insole treatment. With the pressure distributions reported, patients experiencing symptoms in the rearfoot or forefoot may be better suited for WEDG+V-ARCH, since pressure in these affected plantar regions are considerably lower, and less likely to exacerbate symptoms. In contrast, patients experiencing symptoms in the midfoot region may be better suited for WEDG alone, since this insole does not significantly increase midfoot pressure compared to FLAT. Future clinical trial work examining this potential impact on clinical care is needed to confirm these preliminary findings from young, healthy individuals.

Ratings of comfort change relative to FLAT were not significantly different between any of the LWI conditions. These insoles were tested over a period of 10–15 min per insole condition, using a small sample (*n* = 40) of healthy individuals that did not use orthotic insoles habitually. As such, it is unsurprising that average comfort ratings for all LWI ranged between 0 (no change) and − 1 on a − 7 to + 7 scale. Evaluating insole comfort in healthy individuals provides foundational information prior to use in clinical populations that could have concomitant foot pathologies. Future research in the comfort effects of these LWI would benefit from longer study durations and using cohorts of patients with knee OA. As previously mentioned, only WEDG and WEDG+V-ARCH reduced the KAM [12]. Until we know otherwise with data from patients with knee OA, these insoles can perhaps be treated equivalently for their biomechanical efficacy, and the prescription of either LWI should prioritize minimizing regional pressures and improving subjective comfort.

Findings from this study should be interpreted within the context of several limitations. Firstly, healthy adults without a history of orthotic use were recruited for this exploratory study of plantar pressure changes with LWI and supported-LWI, intended for patients with knee OA. While it is imperative future investigations be conducted within the target clinical population to assess the effects on pain and other clinically-relevant outcomes, our findings did not demonstrate any immediate effects of plantar pressure distribution or comfort that would pose any major risk of adverse effects with insole use. Indeed, current clinical guidelines [28] for management of knee OA do not necessarily support the use of LWIs for knee OA – based primarily on contradictory findings of pain improvement [29]. While any biomechanical benefits of these devices are generally not considered in these guideline recommendations, our current findings do provide new evidence that may be used in such decisions. Next, we used flexible in-shoe pressure sensors to measure plantar pressure distribution from a non-planar surface at the foot-insole interface, which may not reflect the CoP measured by floor-mounted force platforms or pressure mats. Indeed, floor-measured data provides CoP data necessary for evaluations of mechanisms of KAM reduction. However, we demonstrated in-shoe pressure sensors are a cost-effective method to obtain regional pressure distribution information that could inform the insole prescription process amidst considerations of existing foot symptoms. Finally, while longer acclimatization periods with insoles are common for clinical practice (~ 2–4 weeks), the number of insole conditions insoles that we tested prevented us from doing so within the current study design. However, participants were encouraged to take as long as they felt was necessary to adjust to the sensation of each new orthotic, prior to collection of gait trials.

## Conclusion

In our exploration of plantar pressure in healthy adults walking with LWI, with or without medial arch support, the data demonstrated pressure distributions that may streamline the selection process for which type of LWI to implement for biomechanical intervention. All LWI shifted the medial-lateral distribution laterally, including the WEDG+U-ARCH which was previously shown to be ineffective at reducing the KAM. Therefore, shifts in foot CoP may not be the primary mechanism by which LWI can reduce the KAM. Regional plantar pressure distributions point towards using the WEDG+V-ARCH for individuals experiencing foot symptoms in the rearfoot and forefoot, and the WEDG for individuals experiencing foot symptoms in the midfoot. Since comfort ratings did not differ between insole conditions, the proposed arrangement of LWI recommendations aims to reduce pressures in the associated plantar region and minimize the chance of exacerbating concomitant foot symptoms in patients with knee OA.

## Supplementary Information

**Additional file 1.**

## Data Availability

The datasets used and/or analyzed during the current study are available from the corresponding author on reasonable request.

## Abbreviations

AUC: Area under curve
CoP: Centre of pressure
EVA: Ethyl vinyl acetate foam
FLAT: 3 mm EVA flat control insole
KAM: Knee adduction moment
Knee OA: Medial tibiofemoral osteoarthritis
kPa: kilopascal, unit of pressure
kPa*sec: product of kilopascal and time, unit of cumulative pressure over time
LWI: Lateral wedge insole
MLPI: Medial-lateral pressure index
PTI: Pressure-time integral
Supported-LWI: Lateral wedge insole with medial arch support
U-ARCH: Uniform-stiffness contoured arch support insole
V-ARCH: Variable-stiffness contoured arch support insole
WEDG: 5° lateral wedge insole
WEDG+U-ARCH: Lateral wedge with uniform-stiffness contoured arch support insole
WEDG+V-ARCH: Lateral wedge with variable-stiffness contoured arch support insole

## References

[1] Chang AH, Moisio KC, Chmiel JS, Eckstein F, Guermazi A, Prasad PV, Zhang Y, Almagor O, Belisle L, Hayes K, Sharma L (2015). External knee adduction and flexion moments during gait and medial tibiofemoral disease progression in knee osteoarthritis. Osteoarthr Cartil.

[2] Miyazaki T, Wada M, Kawahara H, Sato M, Baba H, Shimada S (2002). Dynamic load at baseline can predict radiographic disease progression in medial compartment knee osteoarthritis. Ann Rheum Dis.

[3] Hatfield GL, Stanish WD, Hubley-Kozey CL (2015). Three-dimensional biomechanical gait characteristics at baseline are associated with progression to total knee arthroplasty. Arthritis Care Res.

[4] Shaw KE, Charlton JM, Perry CKL, De Vries CM, Redekopp MJ, White JA (2018). The effects of shoe-worn insoles on gait biomechanics in people with knee osteoarthritis: a systematic review and meta-analysis. Br J Sports Med.

[5] Paterson KL, Hinman RS, Hunter DJ, Wrigley TV, Bennell KL (2015). Impact of concurrent foot pain on health and functional status in people with knee osteoarthritis: data from the osteoarthritis initiative. Arthritis Care Res.

[6] Paterson KL, Kasza J, Hunter DJ, Hinman RS, Menz HB, Peat G, Bennell KL (2017). The relationship between foot and ankle symptoms and risk of developing knee osteoarthritis: data from the osteoarthritis initiative. Osteoarthr Cartil.

[7] Paterson KL, Kasza J, Hunter DJ, Hinman RS, Menz HB, Peat G, Bennell KL (2017). Longitudinal association between foot and ankle symptoms and worsening of symptomatic radiographic knee osteoarthritis: data from the osteoarthritis initiative. Osteoarthr Cartil.

[8] Hatfield GL, Cochrane CK, Takacs J, Krowchuk NM, Chang R, Hinman RS, Hunt MA (2016). Knee and ankle biomechanics with lateral wedges with and without a custom arch support in those with medial knee osteoarthritis and flat feet. J Orthop Res.

[9] Chapman GJ, Parkes MJ, Forsythe L, Felson DT, Jones RK (2015). Ankle motion influences the external knee adduction moment and may predict who will respond to lateral wedge insoles?: an ancillary analysis from the SILK trial. Osteoarthr Cartil.

[10] Tipnis RA, Anloague PA, Laubach LL, Barrios JA (2014). The dose-response relationship between lateral foot wedging and the reduction of knee adduction moment. Clin Biomech.

[11] Kerrigan DC, Lelas JL, Goggins J, Merriman GJ, Kaplan RJ, Felson DT (2002). Effectiveness of a lateral-wedge insole on knee varus torque in patients with knee osteoarthritis. Arch Phys Med Rehabil.

[12] Tse CTF, Ryan MB, Hunt MA (2020). Influence of foot posture on immediate biomechanical responses during walking to variable-stiffness supported lateral wedge insole designs. Gait Posture.

[13] Hunt MA, Takacs J, Krowchuk NM, Hatfield GL, Hinman RS, Chang R (2017). Lateral wedges with and without custom arch support for people with medial knee osteoarthritis and pronated feet: an exploratory randomized crossover study. J Foot Ankle Res.

[14] Sawada T, Tanimoto K, Tokuda K, Iwamoto Y, Ogata Y, Anan M, Takahashi M, Kito N, Shinkoda K (2017). Rear foot kinematics when wearing lateral wedge insoles and foot alignment influence the effect of knee adduction moment for medial knee osteoarthritis. Gait Posture.

[15] Hinman RS, Bowles KA, Metcalf BB, Wrigley TV, Bennell KL (2012). Lateral wedge insoles for medial knee osteoarthritis: effects on lower limb frontal plane biomechanics. Clin Biomech.

[16] Ferrigno C, Wimmer MA, Trombley RM, Lundberg HJ, Shakoor N, Thorp LE (2016). A reduction in the knee adduction moment with medial thrust gait is associated with a medial shift in center of plantar pressure. Med Eng Phys.

[17] Erhart JC, Mündermann A, Mündermann L, Andriacchi TP (2008). Predicting changes in knee adduction moment due to load-altering interventions from pressure distribution at the foot in healthy subjects. J Biomech.

[18] Tarrade T, Doucet F, Saint-Lô N, Llari M, Behr M (2019). Are custom-made foot orthoses of any interest on the treatment of foot pain for prolonged standing workers?. Appl Ergon.

[19] Metcalf B, Paterson KL, Campbell PK, Wrigley TV, Kasza J, Bennell KL, Hinman RS (2019). Relationship between static foot posture, in-shoe plantar forces and knee pain in people with medial knee osteoarthritis. Osteoarthr Cartil.

[20] Van Gheluwe B, Dananberg HJ (2004). Changes in plantar foot pressure with in-shoe varus or valgus wedging. J Am Podiatr Med Assoc.

[21] van Tunen JAC, Paterson KL, Wrigley T V., Metcalf BR, Thorlund JB, Hinman RS. Effect of knee unloading shoes on regional plantar forces in people with symptomatic knee osteoarthritis - an exploratory study. J Foot Ankle Res 2018;11(1):1–8, doi: 10.1186/s13047-018-0278-x. 10.1186/s13047-018-0278-x PMC6019230 29983749

[22] Franz JR, Dicharry J, Riley PO, Jackson K, Wilder RP, Kerrigan DC (2008). The influence of arch supports on knee torques relevant to knee osteoarthritis. Med Sci Sports Exerc.

[23] Hinman RS, Bardin L, Simic M, Bennell KL (2013). Medial arch supports do not significantly alter the knee adduction moment in people with knee osteoarthritis. Osteoarthr Cartil.

[24] Ferrigno C, Stoller IS, Shakoor N, Thorp LE, Wimmer MA (2016). The feasibility of using augmented auditory feedback from a pressure detecting insole to reduce the knee adduction moment: a proof of concept study. J Biomech Eng.

[25] Kamper SJ, Maher CG, MacKay G (2012). Global rating of change scales: a review of strengths and weaknesses and considerations for design. J Man Manip Ther.

[26] The jamovi project (2021). jamovi (Version 1.6) [Computer Software].

[27] Hunt MA, Charlton JM, Felson DT, Liu A, Chapman GE, Graffos A (2021). Frontal plane knee alignment mediates the effect of frontal plane rearfoot motion on knee joint load distribution during walking in people with medial knee osteoarthritis. Osteoarthr Cartil.

[28] Kolasinski SL, Neogi T, Hochberg MC, Oatis C, Guyatt G, Block J, Callahan L, Copenhaver C, Dodge C, Felson D, Gellar K, Harvey WF, Hawker G, Herzig E, Kwoh CK, Nelson AE, Samuels J, Scanzello C, White D, Wise B, Altman RD, DiRenzo D, Fontanarosa J, Giradi G, Ishimori M, Misra D, Shah AA, Shmagel AK, Thoma LM, Turgunbaev M, Turner AS, Reston J (2020). 2019 American College of Rheumatology/Arthritis Foundation guideline for the Management of Osteoarthritis of the hand, hip, and knee. Arthritis Care Res.

[29] Parkes MJ, Maricar N, Lunt M, LaValley MP, Jones RK, Segal NA (2013). Lateral wedge insoles as a conservative treatment for pain in patients with medial knee osteoarthritis a meta-analysis. JAMA.

