# Supplementary material for: An exploration of changes in plantar pressure distributions during walking with standalone and supported lateral wedge insole designs
Source: J Foot Ankle Res. 2021 Oct 6;14:55. doi: 10.1186/s13047-021-00493-5 (PMC8493692; doi:10.1186/s13047-021-00493-5)
Supplement: Supplementary file 1 — Additional file 1. [file 13047_2021_493_MOESM1_ESM.docx]

**Appendix Table 1.** Comfort and medial-lateral pressure index (MLPI) outcomes by insole condition.

| **Outcome** | | **FLAT** | **WEDG** | **V-ARCH** | **U-ARCH** | **WEDG +**  **V-ARCH** | **WEDG +**  **U-ARCH** |
| --- | --- | --- | --- | --- | --- | --- | --- |
|  | Comfort Change  (-7 to + 7) | – | -1.2 (2.3) | 0.0 (2.3) | -0.6 (2.9) | -0.8 (2.2) | -0.7 (2.5) |
| Early Stance | Mean  (% foot width) | 3.2 (2.9) | 5.5 (2.5) | 5.0 (3.3) | 5.7 (3.0) | 5.8 (3.0) | 6.9 (3.0) |
|  | AUC  (% foot width * sec) | 1.0 (0.9) | 1.8 (0.8) | 1.6 (1.1) | 1.9 (1.0) | 1.9 (1.0) | 2.3 (1.0) |
| Late Stance | Mean  (% foot width) | -2.5 (5.7) | 0.5 (6.0) | -3.1 (6.9) | -1.8 (6.4) | -0.4 (5.9) | 0.9 (6.2) |
|  | AUC  (% foot width * sec) | -0.8 (1.9) | 0.2 (2.0) | -1.0 (2.3) | -0.6 (2.2) | -0.1 (2.0) | 0.4 (2.1) |

All values reported as mean (standard deviation).

Positive (lateral) and negative (medial) MLPI values indicate the centre of pressure position relative to the midline of the foot.

**Appendix Table 2.** Regional plantar pressure outcomes by insole condition, reported as mean (standard deviation).

| **Plantar Region** | **Pressure Outcome** | **FLAT** | **WEDG** | **V-ARCH** | **U-ARCH** | **WEDG +**  **V-ARCH** | **WEDG +**  **U-ARCH** |
| --- | --- | --- | --- | --- | --- | --- | --- |
| Lateral Rearfoot | Peak Pressure  (kPa) | 133.0 (24.1) | 132.6 (26.0) | 122.3 (27.8) | 120.4 (22.4) | 122.0 (22.6) | 126.1 (23.4) |
|  | Pressure-Time Integral (kPa*sec) | 30.0 (6.4) | 29.0 (5.3) | 26.9 (8.4) | 26.6 (5.6) | 26.7 (5.5) | 28.3 (6.9) |
|  | Contact Area  (cm^2^) | 18.7 (2.3) | 18.1 (2.1) | 19.8 (2.3) | 19.8 (2.4) | 19.5 (2.2) | 19.9 (2.6) |
| Lateral Forefoot | Peak Pressure  (kPa) | 106.1 (31.8) | 108.6 (33.3) | 75.4 (30.9) | 87.0 (27.3) | 85.6 (26.7) | 100.7 (32.2) |
|  | Pressure-Time Integral (kPa*sec) | 30.6 (10.4) | 32.4 (10.7) | 18.1 (8.7) | 22.1 (7.3) | 22.0 (7.3) | 26.8 (10.1) |
|  | Contact Area  (cm^2^) | 12.9 (2.6) | 12.6 (2.3) | 12.3 (2.2) | 13.0 (1.9) | 12.2 (2.2) | 12.3 (2.2) |
| Medial Rearfoot | Peak Pressure  (kPa) | 150.3 (23.2) | 131.9 (26.1) | 100.9 (25.1) | 94.9 (25.7) | 105.3 (27.6) | 99.3 (21.5) |
|  | Pressure-Time Integral (kPa*sec) | 32.9 (6.7) | 26.6 (5.8) | 21.0 (5.9) | 19.9 (6.1) | 21.4 (6.1) | 20.4 (5.2) |
|  | Contact Area  (cm^2^) | 17.5 (2.1) | 16.5 (2.1) | 18.2 (2.4) | 17.7 (2.7) | 17.9 (2.5) | 17.9 (2.8) |
| Medial Forefoot | Peak Pressure  (kPa) | 125.3 (43.0) | 105.4 (39.6) | 106.4 (41.2) | 101.4 (39.5) | 96.9 (34.2) | 93.4 (37.0) |
|  | Pressure-Time Integral (kPa*sec) | 30.1 (10.3) | 23.4 (8.6) | 22.5 (8.9) | 20.4 (8.7) | 20.2 (7.3) | 18.3 (7.6) |
|  | Contact Area  (cm^2^) | 14.4 (1.8) | 13.9 (2.1) | 15.0 (2.6) | 15.0 (2.8) | 14.5 (2.6) | 14.7 (2.6) |
| Midfoot | Peak Pressure  (kPa) | 42.8 (11.8) | 45.6 (10.6) | 43.6 (10.9) | 43.0 (11.0) | 48.1 (12.9) | 48.0 (12.9) |
|  | Pressure-Time Integral (kPa*sec) | 14.1 (4.6) | 15.5 (4.7) | 14.5 (4.3) | 14.2 (4.2) | 16.2 (4.8) | 16.2 (5.2) |
|  | Contact Area  (cm^2^) | 34.7 (8.7) | 31.8 (7.7) | 52.0 (9.9) | 53.0 (10.2) | 50.9 (9.3) | 52.8 (9.1) |

All values reported as mean (standard deviation).
